# The RNA exosome: mechanisms of RNA surveillance, regulation, and disease

**DOI:** 10.1080/15476286.2026.2685379

**Published:** 2026-06-11

**Authors:** Priyanka Upadhyai, Neha Quadri, Divya Chandran

**Affiliations:** a Department of Medical Genetics, Kasturba Medical College, Manipal Academy of Higher Education, Manipal, India; b Centre for Molecular Neurosciences, Department of Anatomy, Kasturba Medical College, Manipal Academy of Higher Education, Manipal, India

**Keywords:** RNA exosome, RNA exosome cofactors, RNA exosomopathies, RNA surveillance, RNA quality control

## Abstract

The RNA exosome is a conserved multi-subunit ribonuclease complex with pivotal roles in RNA biogenesis, surveillance, and processing. It comprises a nine-subunit scaffold that associates with distinct ribonucleases in a cell compartment-specific manner, contributing to the processing and turnover of a broad spectrum of nuclear and cytoplasmic transcripts, including pervasively transcribed and short-lived RNAs, precursors, and abortive and aberrant transcripts. In this review, we examine how the RNA exosome engages a wide spectrum of RNAs via differential adaptor usage and intrinsic substrate features, such as transcript length and 3’ end structure. This also modulates the entry routes of the recruited transcripts. We highlight conserved principles and major differences between yeast and metazoans. We assimilate emerging evidence that suggests that the RNA exosome localization and activity are dynamically regulated in response to cellular context and external stimuli. Finally, drawing on findings from studies in *S. cerevisiae, Drosophila*, zebrafish, and mice, we discuss how perturbations in RNA surveillance can result in abnormalities in organismal development and homoeostasis. Together, these studies not only enhance our knowledge of the broader relevance of RNA quality control and metabolism but also provide mechanistic insights into pathomechanisms, particularly the tissue-specific vulnerabilities noted in RNA exosome-linked diseases.

## 1. Introduction

The RNA exosome is a conserved multi-subunit complex, which acts as a pivotal regulatory node in RNA fate determination, operating at the crossroads of transcription, RNA processing, and translation. It modulates the turnover and processing of a plethora of canonical noncoding RNAs (ncRNAs), such as snRNAs, snoRNAs and tRNAs, as well as, short-lived ncRNAs from intergenic regions [1–3] that are produced due to a widespread propensity for pervasive transcription by RNA Polymerase II (Pol II) [4] in the nucleus. In the cytoplasm, it contributes to the degradation of improperly processed and spurious mRNAs [5], as well as hypomodified tRNAs [6]. First identified in *Saccharomyces cerevisiae* as a ribonucleolytic complex required for rRNA processing and maturation [7], the RNA exosome is composed of a nine-subunit central barrel (EXO9) that recruits nucleases in a cell compartment-specific manner. In budding yeast, its subunits are termed as ribosomal RNA processing (Rrp) proteins [7]; in humans, they are referred to as exosome components (EXOSC). The functionality of the RNA exosome derives from its modular design, that is amplified by adaptors conferring substrate specificity, delineating access routes to distinct catalytic subunits and decision-making between turnover and trimming of transcripts.

The physiological relevance of the RNA exosome is underscored by a growing class of human diseases caused by its dysfunction termed collectively as RNA exosomopathies, which manifest with overlapping but sometimes distinct phenotypes alluding to tissue-specific differences in vulnerabilities to perturbations in RNA surveillance. Mechanistic insights from *in vivo* models also suggest that each exosome subunit contributes uniquely to RNA metabolism, such that their defects alter the transcriptome in overlapping but markedly distinct ways, thereby resulting in largely unique developmental and physiological outcomes.

In this review, we assimilate the recent advances in RNA exosome biology, integrating findings pertaining to mechanisms of how it recruits and degrades distinct substrates differently with the updated knowledge of how distinct adaptor complexes assist the exosome during nuclear and cytoplasmic RNA-targeting. We discuss how the exosome is itself regulated through compartment-specific localization and post-translational control. Finally, we examine the growing spectrum of human disorders caused by exosome dysfunction, drawing on findings from budding yeast, *Drosophila*, zebrafish, and mouse to illustrate how individual subunits may play unique roles in sculpting the transcriptome during *in vivo* developmental and physiological programmes.

## 2. Architecture of the RNA exosome

### 2.1. RNA exosome core and catalytic components

**Figure 1.**
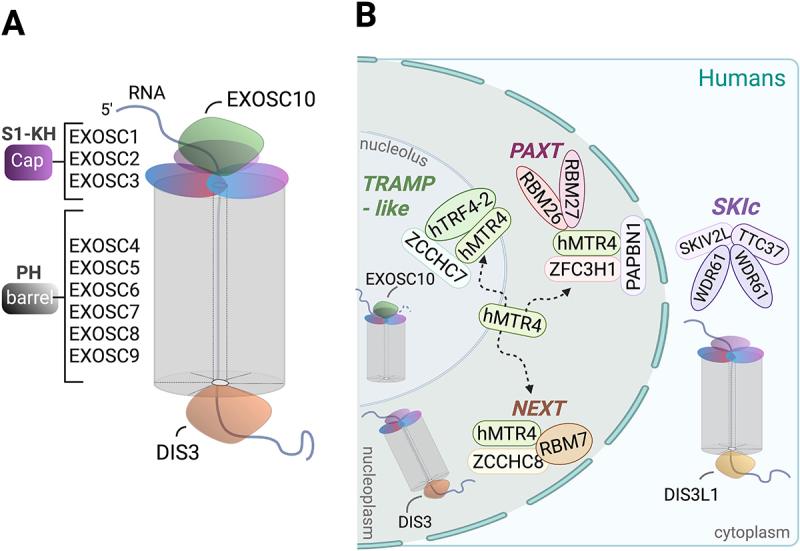
**Architecture of the human RNA exosome and its cofactors. A**. schematic representation of the RNA exosome that comprises a nine-subunit scaffold (EXO9): a six-subunit PH-domain containing barrel (EXOSC4-EXOSC9) with a central channel through which a single RNA substrate can be threaded and capped by three S1-KH-domain-containing subunits (EXOSC1-EXOSC3). This associates with distinct 3’-5’ nucleases: the exoribonuclease EXOSC10 binds the cap, DIS3 with endo- and exoribonuclease, and DIS3L1 with exoribonuclease, that associate with the base of EXO9, in a mutually exclusive manner. **B**. RNA exosome cofactor assemblies and catalytic subunits with their subcellular localization. EXOSC10, DIS3 and DIS3L1 preferentially associate with EXO9 in the nucleolus, nucleoplasm and cytoplasm, respectively. The RNA helicase MTR4 is shared across several distinct nuclear exosome cofactor assemblies in a mutually exclusive manner: in the nucleolus it combines with ZCCHC7 and hTRF4-2 to form the human TRAMP-like complex; in the nucleoplasm it is incorporated in the NEXT and PAXT adaptor complexes by binding ZCCHC8 and ZFC3H1 RNA-binding proteins, respectively. In the cytoplasm the SKI complex is composed of SKIV2L, TTC37 and WDR61 that are orthologous to the budding yeast ySki2, ySki3, and ySki8 and associate with the exosome for its function.

Initial studies resolved the structure of *S. cerevisiae* and human EXO9 comprising a donut-shaped central ring composed of six subunits containing RNase PH-like domains: yRrp41/EXOSC4, yRrp46/EXOSC5, yMtr3/EXOSC6, yRrp42/EXOSC7, yRrp43/EXOSC8, and yRrp45/EXOSC9, together forming a complex [8,9]. The EXO9 central barrel is capped by a trimeric lid comprising yCsl4/EXOSC1, yRrp4/EXOSC2 and yRrp40/EXOSC3, which contributes to transcript engagement (Figure 1). yRrp4 and yRrp40 each contain an N-terminal domain, followed by middle S1 and C-terminal KH RNA-binding domains. yCsl4 harbours a central S1 domain with adjoining N- and C-terminal regions. Structural studies in budding yeast uncovered that the ribonucleases yRrp6/EXOSC10 and yRrp44/DIS3 associate with EXO9 at its top and bottom, respectively (Figure 1). The EXO9 central barrel contains an 8–10 Å wide cavity at its core that can accommodate a single strand of the RNA substrate [8,10] threaded through it for delivery to the catalytic subunit [9,11,12].

The budding yeast yRrp44 is a member of the RNAse II/R family [13]. It comprises a central 3’-5’ exoribonuclease RNB domain flanked by an S1 and two cold shock domains, as well as harbours an N-terminal endoribonuclease PIN domain [14–17]. Unlike in yeast, humans harbour DIS3 and DIS3-like exoribonuclease 1 (DIS3L1) enzymes [18,19], with DIS3L1 functioning exclusively as an exoribonuclease.

yRrp6/EXOSC10 is a member of the DEDD nuclease superfamily [20]. Its structure was resolved in association with the RNA exosome, cofactors and substrates in *S. cerevisiae* [9,11,21] and humans [22,23]. It harbours a polycystin 2 N-terminal (PMC2NT) domain that interacts with yRrp47 or C1D that function to stabilize it and assist in RNA exosome cofactor recruitment [24–26]. The central region of yRrp6 contains an exoribonuclease active site and the regulatory helicase and RNase D C-terminal (HRDC) domain, which interacts with the catalytic site to regulate its activity [21]. The C-terminal region of yRrp6 is unstructured when not associated with the exosome but upon its association with yCsl4, yRrp43 and yMtr3 via their PH-ring-like regions it forms the exosome associating region (EAR) domain [9,11,27]. A stretch of 100 residues in the yRrp6 C-terminal tail termed the ‘lasso’ is basic, unstructured and contains a nuclear localization sequence, binding substrate and activating the RNA exosome [28].

### 2.2. Cofactors

Given that the RNA exosome is ubiquitous and the EXO9 core is highly conserved in terms of overall architecture, the exosome shows context-dependent conformational flexibility, relying on a repertoire of auxiliary factors for substrate selection, and decision-making between degradation and processing. Four cofactor complexes, discussed below, modulate its function and adaptability as the transcriptional landscape is dynamically altered during development, morphogenesis and homoeostasis. The trimeric Trf4-Air2-Mtr4 Polyadenylation (TRAMP) complex is one of the well-known exosome cofactors operating in the nucleus in *S. cerevisiae* [29–31]. It comprises yMtr4, a 3’-5’ RNA helicase, yTrf4, a non-canonical poly(A) polymerase and yAir2, an RNA-binding protein. Using mass spectrometry in budding yeast at least three distinct subsets of TRAMP assemblies with nonredundant roles have been discovered, where differential substrate specificities are conferred by yTrf4 and its paralog yTrf5 [32]. In humans, a TRAMP-like assembly composed of MTREX/SKIV2L2/MTR4, Poly(A) RNA Polymerase D5 (hPAPD5) and Zinc finger CCHC-domain containing protein 7 (hZCCHC7) predominantly functions in the nucleolus [33] (Figure 1B). Crystallographic studies of yeast TRAMP showed that yMtr4 is tethered to yRrp6-yRrp47 via its N-terminal domain [25]. It is unclear whether this interaction is conserved; however, cryo-electron microscopy analysis of human nuclear exosomes bound to MTR4 have revealed that the helicase is docked at the EXO9 cap through direct contact with EXOSC2, with MPP6 bridging this interface by linking MTR4 to EXOSC1 and EXOSC3 [23,27].

In addition, to the TRAMP, human MTR4 is incorporated into distinct cofactor assemblies by binding different RNA-binding proteins. This is illustrated by X-ray crystallographic and nuclear magnetic resonance experiments that showed that adaptor subunits, such as the zinc-finger proteins, ZCCHC8 and ZFC3H1 interact with MTR4 through unique ‘arch’ domain-interacting motifs and recruit it into the Nuclear Exosome Targeting (NEXT) and Poly(A) tail eXosome Targeting (PAXT) complexes located in the nucleoplasm [34] (Figure 1B). NEXT is a dimer of a heterotrimer containing hMTR4, RBM7, an RNA-binding protein and ZCCHC8 [33,35]. PAXT includes ZFC3H1 and ZC3H3, another zinc-finger protein that regulates mRNA adenylation and export from the nucleus [36], RBM26/27, RNA-binding proteins and the nuclear poly(A)-binding protein (PABPN1) that accumulate in nuclear foci [37], thereby providing a scaffold to engage the exosome and gatekeep its substrate RNAs.

In the cytoplasm, the RNA exosome associates with the conserved Ski2-Ski3-Ski8 (Ski) complex, consisting of ySki2, the DExH-box helicase, ySki3, a tetratricopeptide repeat protein and WD-repeat containing ySki8; in humans this complex is assembled from their orthologues SKIV2L, TTC37 and WDR61 (Figure 1B), respectively, and reviewed extensively elsewhere [38]. The yeast Ski7p is a GTP-binding adaptor that bridges the Ski complex to the cytoplasmic exosome [39,40] and an analogous role is performed by the short isoform of HBS1L3 in humans. High-resolution structural studies in budding yeast [41] and humans [42] have elucidated how conformational rearrangements of the Ski complex enable RNA substrate threading through the exosome to coordinate with ribosome-associated surveillance pathways.

## 3. RNA exosome substrates, adaptors and entry-routes

**Figure 2.**
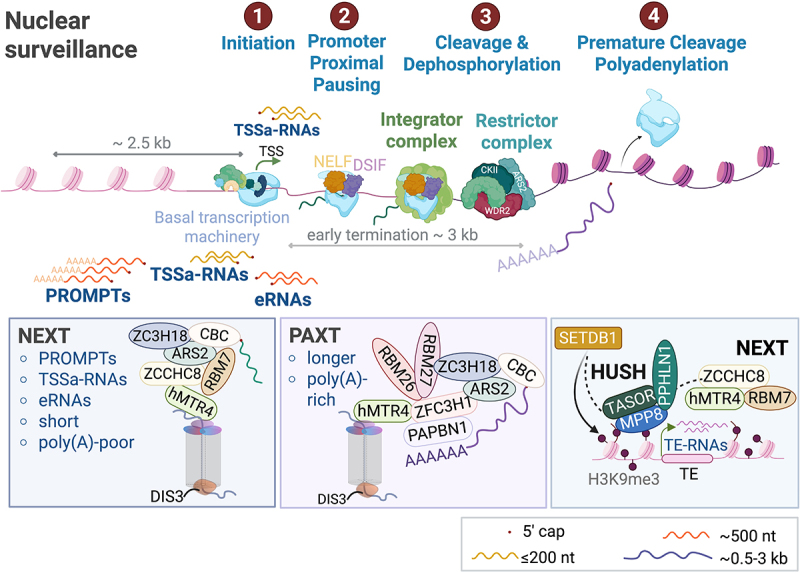
**Overview of RNA exosome adaptor usage for targeting varied RNA species**. Nuclear surveillance monitors pervasively transcribed short-lived RNAs, such as PROMPTs, TSSa-RNAs, and eRNAs, arising from the TSS and promoter-proximal paused regions. Early transcription termination events mediated by the Integrator and Restrictor complexes also result in unstable RNA species, while PCPA generates longer and more polyadenylated RNAs. Different RNA species are selectively recognized by distinct adaptor pathways engaged with the RNA exosome. The NEXT complex (hMTR4–ZCCHC8–RBM7), together with the CBC and ARS2, targets short, capped, and poly(A) deficient transcripts, such as PROMPTs, TSSa-RNAs, and eRNAs. The PAXT machinery (hMTR4–ZFC3H1–RBM26/27–PABPN1) predominantly targets longer, and more polyadenylated nuclear RNAs. In addition, transcripts derived from genomic regions enriched in transposable elements (TE) or TE-RNAs are decayed through the HUSH complex (TASOR-PPHLN1-MPP8), which can interface with NEXT-mediated exosome recruitment, at transcriptional and post-transcriptional stages. HUSH is recruited to TE loci enriched with H3K9me3 repressive histone modifications by SETDB1. NEXT is engaged by HUSH through the interaction between ZCCHC8 and MPP8.

Eukaryotic transcription is inherently pervasive, producing a wide spectrum of transcripts that extend beyond well-annotated protein-coding RNAs and ncRNAs with defined catalytic and regulatory roles [43]. Most pervasive transcripts are highly unstable and do not accumulate to detectable levels owing to their rapid degradation by the nuclear RNA surveillance pathways, among which the RNA exosome plays a focal role in transcript surveillance, turnover, and processing (Figure 2). In contrast, bulk cytoplasmic mRNA turnover predominantly proceeds via the XRN1-dependent machinery, and RNA surveillance occurs in a context-dependent manner, with XRN1 mediating 5′–3′ decay and SKI-exosome mediating 3′–5′ degradation, including in translation-coupled quality control pathways.

### 3.1. Nuclear exosome RNA targets

#### 3.1.1. Pervasive transcription

Early evidence of short, unstable transcripts emerged in *S. cerevisiae*, where 5’ capped and 3’-end polyadenylated ncRNAs termed cryptic unstable transcripts (CUTs) that are degraded by the TRAMP-nuclear exosome pathway were identified [31,44,45]. Subsequent transcriptomic work has revealed that pervasive transcription is widespread among eukaryotes, with a substantial fraction of the mammalian genome being pervasively transcribed [43], not only from intergenic regions but also from divergent promoters and enhancers. Pol II generates a spectrum of unstable ncRNAs from the vicinity of active promoters of protein-coding genes. These include proximal, short (~20–200 nt), capped and non-polyadenylated sense and antisense transcripts, termed transcription start site (TSS)-associated RNAs (TSSa-RNAs) [46,47], which arise from ±400bp of the TSS. Additional classes of short-lived RNA species include longer (~450 nt), antisense, capped and polyadenylated promoter upstream transcripts (PROMPTs) [1,48,49], also termed upstream antisense RNAs (uaRNAs) [50] arising within 0.5–2.5 kb of the TSS. Active enhancers generate short-lived, bidirectional enhancer RNAs (eRNAs) that are typically capped, unspliced, and predominantly non-polyadenylated, with most being short (~500 nt), although occasionally they can be substantially longer (>1–5 kb) [49,51]. Divergent promoter transcription and eRNA production are widespread in mammals but are rarer among less complex metazoans, such as *Drosophila* [52]. A contributor to these pervasive transcripts is the inherent tendency of Pol II to initiate transcription from any accessible genomic region, such as those enriched with CpG islands or depleted of nucleosomes [4,53]. Studies in yeast and humans have uncovered additional short-lived ncRNAs that are partially non-polyadenylated and arise due to constitutive readthrough transcription termed downstream of gene-containing transcripts (DoGs) [54], upstream of gene transcripts (UoG) [55], as well as transcripts initiated from cryptic promoters within gene bodies [55–57].

Our understanding of the biological significance of pervasive ncRNAs is still evolving. TSSa-RNAs are integral to the general mammalian transcription landscape and reflect regions of Pol II pausing at actively transcribed promoters [47,53]. DoG production is triggered not only in response to stress [58] but also represents a widespread, non-stochastic mechanism of gene expression regulation under native conditions [59]. Intergenic RNAs associated with annotated genes have been postulated to exert context-specific local effects [60]. PROMPTs are implicated in specific transcriptional contexts, for example during the activation of oestrogen-responsive genes via upregulation of 7SK-P-TEFb complex [61]. eRNAs not only function in cis for modulation of gene expression at adjacent loci but also exert regulatory roles in *trans*, as reviewed elsewhere [62].

These classes of unstable nuclear transcripts are recruited for exosome-mediated decay in a cofactor dependent manner. While NEXT targets short-lived RNA species, such as eRNAs, PROMPTs and others that are non-polyadenylated or poorly polyadenylated [63], PAXT preferentially degrades longer and highly polyadenylated RNA substrates [64] (Figure 2).

#### 3.1.2. Premature transcription products

While Pol II is envisioned as a highly processive enzyme, a substantial fraction of promoter-proximal Pol II complexes undergoes premature termination generating unstable transcriptional byproducts [65,66]. Promoter-proximal Pol II pausing is facilitated by negative elongation factor (NELF) and DRB-sensitivity inducing factor (DSIF), which establish an early regulatory checkpoint distinguishing the early phase of transcription from productive elongation. Transcriptional elongation requires the positive transcription elongation factor b (P-TEFb), which mediates the transition of the basal transcription machinery beyond this checkpoint [67]. Pol II pausing is spatiotemporally modulated [68] and generates short unstable RNA byproducts, such as TSSa-RNAs at many developmentally regulated genes [69,70]. The +1 nucleosome downstream of the TSS acts as a steric barrier, reinforcing Pol II pausing and increasing the production of transient abortive transcripts in promoter-proximal regions [71,72]. In addition, the trimethylation of histone H3 lysine 4 trimethylation (H3K4me3) regulates Pol II pause release and productive elongation [73].

The Integrator complex, best known for its canonical role in 3’ end processing of snRNAs, associates with the C-terminal domain of Pol II at paused promoters to mediate premature transcription termination, thereby generating short, capped and poorly or non-polyadenylated transcripts [74–76]. Following pause release, Pol II enters an early ‘restriction’ zone, which constitutes a more global transcription checkpoint where the elongation of ncRNAs is curtailed by the Restrictor complex, composed of ZC3H4 with WD-repeat factor WDR82 [77–80].

Another mechanism of premature transcription termination is premature cleavage polyadenylation (PCPA), which frequently occurs at cryptic polyadenylation sites (PASs) within both protein-coding and noncoding regions, as well as at promoters producing divergent antisense transcripts [81]. Given that PAS motifs occur frequently (~90%) in the intronic regions of human genes [82], PCPA is normally averted by U1 small nuclear ribonucleoprotein (U1 snRNP) binding to upstream canonical or cryptic splice donor sites [83,84], a process termed telescripting. Productive mRNA synthesis is promoted by depletion of PAS motifs across transcription units and enrichment of 5’ splice site (ss) proximal to the TSS, which recruit U1 snRNP to suppress neighbouring PAS usage. PCPA frequently occurs at PAS motifs enriched near Pol-II pause sites, which often coincide with stable nucleosomes within the first few introns of downstream genes. The nascent unstable, capped, and poorly polyadenylated transcripts [75] generated by Integrator-mediated termination are recruited by the cap binding complex (CBC), which binds the ZC3H18 subunit of the NEXT complex via the ARS2 adaptor and channel them for exosome mediated degradation [85] (Figure 2). In contrast, during PCPA U1 associates with cleavage and polyadenylation factors (CPAFs) to generate extensively polyadenylated RNAs, which are preferentially targeted to the exosome by PAXT and to a lesser extent by the NEXT complex [86].

Early mRNA termination in human cells can also be prevented by U1 independent anti-termination mechanisms, which involve proteins such as SCAF4 and SCAF8, but there is currently no evidence for their interaction with the RNA exosome [87].

#### 3.1.3. Aberrant transcripts

The nuclear exosome targets aberrant mRNAs that may have retained introns owing to improper splicing [88,89]. In budding yeast, surveillance of improperly spliced pre-mRNAs involves two shuttling serine/arginine (SR)-like proteins, Gbp2 and Hrb1 that bind to pre-mRNAs and interact with the spliceosome to monitor the fidelity of splicing and prevent the export of aberrant mRNAs [90]. TRAMP and Rrp6 are co-transcriptionally recruited to nascent, improperly spliced transcripts, ensuring rapid degradation of aberrant precursor transcripts [88,91]. Whether an analogous surveillance pathway operates in more complex organisms is unclear.

#### 3.1.4. Ribosomal RNA

Ribosome maturation and assembly involve pre-rRNA processing concomitant with the action of many *trans*-acting factors. The large ribosomal subunit (60S) contains 25S (yeast)/28S (human) rRNA, 5.8S rRNAs and the 5S rRNA that are independently synthesized by RNA Polymerase III; the small subunit (40S) contains 18S rRNA [92,93]. Three rRNAs are co-synthesized in the form of the rRNA precursor transcript termed 35S (budding yeast)/47S (human), which harbours the 18S, 5.8S and 25S/28S rRNAs in a 5’-3’ orientation, flanked by 5’ and 3’ external transcribed spacers (5’/3’ETS) and internal transcribed spacers 1 and 2 (ITS1 and ITS2). The removal of the spacers occurs by a series of well-defined endoribonucleolytic cleavages and exoribonucleotytic trimming steps that allow quality control and regulation. The yeast and human pre-rRNA processing pathways have been described in detail in excellent reviews [94,95].

During large ribosomal subunit assembly, the RNA exosome is involved in 3’ end maturation of the 5.8S rRNA. Following ITS2 cleavage, the 7S pre-rRNA intermediate undergoes progressive trimming by yRrp44/DIS3 followed by final 3’ end maturation by yRrp6/EXOSC10 to generate the mature 5.8S rRNA [7,96–98]. Structural and biochemical insights reveal that pre-rRNA intermediates are channelled through the exosome core to the exonuclease active site of yRrp44 but may also be cleaved by its PIN-domain endonuclease activity [8,9,14]. Recruitment of the RNA exosome to pre-60S particles is facilitated by yMtr4 interacting with the adaptor yNop53 [34,99], while yRrp6 and yRrp47 form a composite surface that stabilizes yMtr4 association with the nuclear exosome [25].

During 18S rRNA maturation and small ribosomal subunit assembly, the small subunit processome (SSU) [100], a large ribonucleoprotein complex assembles co-transcriptionally on the pre-rRNA to catalyse endonucleolytic cleavages that release the 5’-ETS and ITS1, which are subsequently degraded. In yeast, the rRNA processing and degradation of excised spacers are mediated by the coordinated action of TRAMP-exosome, predominantly via yRrp6 [29,30]. Consistent with this, structural and biochemical analyses have shown that the RNA exosome associates with early ribosome biogenesis intermediates, including the 90S pre-ribosome, and mediates yMtr4-dependent processing and degradation of spacer fragments during the pre-40S small ribosomal subunit maturation [101]. In human cells, exosome subunits exhibit functional specialization, with nucleolar EXOSC10 primarily mediating early 5’-ETS degradation, while DIS3 plays a more prominent role in the later processing steps [102].

### 3.2. Mechanistic basis of nuclear RNA targeting to the exosome

#### 3.2.1. TRAMP-dependent decay

In budding yeast, pervasive transcripts such as CUTs are degraded through the co-transcriptional association with the Nrd1–Nab3–Sen1 (NNS) complex, which in turn recruits the TRAMP complex and the RNA exosome [45]. Nuclear transcriptome quality control studies have shown that the yTrf4-dependent non-canonical oligoadenylation adds short poly(A) stretches that serve as destabilization tags [29–31], while yMtr4 remodels structured regions of target transcripts to facilitate exosome-dependent turnover [103]. This pathway preferentially targets unstable RNA for threading through EXO9 central channel for yRrp44-mediated degradation [10,104]. Biochemical studies showed that TRAMP polyadenylates both stable and unstable RNAs, however the poly(A) tails on the former but not the latter are trimmed by yRrp6 aiding in substrate discrimination. The less stable polyadenylated RNAs potentially bypass yRrp6 and are translocated in a Mtr4-dependent manner through the exosome channel for yRrp44-mediated decay [105].

#### 3.2.2. NEXT-linked degradation

In mammals, the NEXT complex, acting through RBM7, targets short-lived RNAs, including 3’-extended precursor transcripts from snRNAs and replication-dependent histone genes, as well as long ncRNAs (lncRNAs), without the prerequisite of polyadenylation [33,63]. PCPA-derived premature transcripts and many PROMPTs remain unspliced, which dissuades their efficient 3’ polyadenylation, thereby impairing their stability and stimulating exosome mediated RNA decay. Degradation of these short ncRNAs may be favoured owing to the close proximity of their 3’ ends with the 5’ cap-bound CBC, which associates with the NEXT-exosome assembly [85].

In mouse embryonic stem cells (mESCs), hMTR4 and ZCCHC8 subunits of NEXT bind to the Microprocessor complex, best known for microRNA (miRNA) processing, to form a variant of NEXT (vNEXT) complex that aids in primary miRNA maturation at super-enhancer [106], and this assembly has been reported to function in the decay of structured eRNAs [107]. Repetitive elements associated with heterochromatin, including ribosomal DNA repeats, centromeres and transposable elements, also produce short and unstable transcripts [63,108] that are degraded by the nuclear exosome. Transcriptional silencing at these repetitive regions is facilitated by the Human Silencing Hub (HUSH) complex, composed of the chromodomain containing protein MPP8, along with TASOR and periphilin (PPHLN1) [109]. The HUSH complex is recruited to repetitive loci delineated by enrichment of H3K9me3 repressive histone marks via H3K9 methyltransferase SETDB1, and in turn recruits the NEXT-exosome through direct interaction of MPP8 and ZCCHC8 [110] (Figure 2).

#### 3.2.3. PAXT-based RNA decay

To facilitate PAXT-exosome activity the poly(A) polymerase γ (PAPγ), a canonical mammalian polymerase that catalyses template independent poly(A) extension at 3’ end of RNAs is recruited to the TSSs of hundreds of human genes in a ZFC3H1-dependent manner. It adds short poly(A) extensions that serve as molecular marks for PAXT-mediated recruitment by the exosome and subsequent decay [111]. This triggers the degradation of diverse ncRNAs including the short-lived PROMPTS/uaRNAs, eRNAs and other prematurely truncated RNAs [64,112], and precursor transcripts such as primary miRNAs and pre-snoRNA via polyA binding protein nuclear 1 (PABPN1) [113,114] and the PAXT machinery [111]. Work in fission yeast has shown that pab2, an ortholog of PABPN1, is involved in polyA trimming of pre-snoRNAs [115].

Misprocessed precursor mRNAs such as those containing intronic PAS and harbouring intact 5’ ss are retained in the nucleus by U1-70K, a U1snRNP component [116], which in turn is targeted via ZFC3H1 for PAXT-exosome degradation [117]. The nuclear degradation of spurious RNA species is reinforced by N6-methyladenosine (m6A) marks, which are recognized by YTH-domain-containing human proteins, YTHDC1 and YTHDC2 that interact with ZFC3H1 promoting PAXT recruitment [118]. Soles *et al.*, 2025 envisioned a nuclear RNA degradation code for prematurely truncated transcripts containing intronic PASs, where the combinatorial loading of the 5’ ss and poly(A) junction but not either alone, along with U1 snRNP and CPA factors promotes the recruitment of PAXT via ZFC3H1 to stimulate exosome dependent decay [119].

### 3.3. Nuclear RNA exosome entry routes

**Figure 3.**
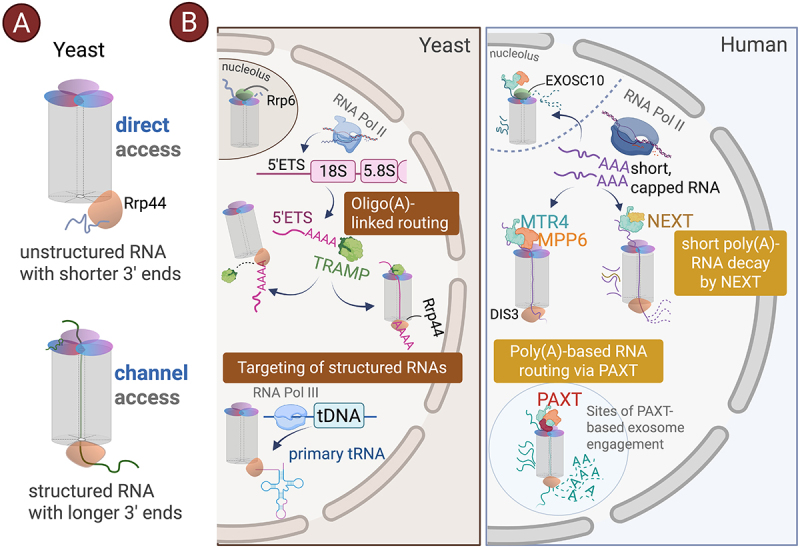
**RNA exosome entry routes and mechanisms of adaptor and nuclease engagement**. Schematic overview of mechanisms governing RNA substrate access to the exosome in budding yeast and humans. A. alternate RNA exosome entry routes (based on budding yeast): unstructured or less constrained transcripts with short 3’ extensions preferentially access yRrp44 ribonuclease directly, and structured substrates with longer 3’-tails are predominantly threaded through the EXO9 central channel to yRrp44. B. in addition to RNA features, adaptor also engagement influences exosome entry in budding yeast. During rRNA maturation, 5’ external spacer (5’ ETS) is co-transcriptionally excised from the pre-rRNA, it is oligoadenylated by TRAMP and preferentially threaded through the EXO9 channel to yRrp44, following which it is released from the channel, undergoes oligoadenylation by TRAMP again and targeted to yRrp44 directly. In parallel, structured RNA substrates, such as those arising by RNA Pol III transcription, such as primary tRNAs, are biased for yRrp44-exosome-mediated decay. In humans, unstable, capped transcripts RNA species, such as those generated by RNA Pol II are routed to the exosome depending on polyadenylation status: poly(A) poor RNAs are primarily recruited by NEXT, threaded through EXO9 channel and degraded by DIS3 in the nucleoplasm; a minority of these transcripts are degraded by EXOSC10 in the nucleolus; poly(A) enriched transcripts are recruited by MPP6-bound exosomes that engage PAXT and enhance delivery to DIS3 by channel-mediated translocation, in discreet PAXT-dependent foci.

Structural and biochemical studies in yeast have revealed that different RNA substrates reach the exosome-linked yRrp44 via alternative entry routes, either through the central channel of the EXO9 core or bypassing it via direct access to yRrp44 [9,10,27,120] (Figure 3). While structured RNAs with longer single-stranded 3’ extensions are preferentially threaded via the channel and induce a conformational change in yRrp44, largely unstructured substrates with shorter 3’ ends access yRrp44 directly [121,122] (Figure 3A).

These findings were confirmed *in vivo*, in budding yeast, the inherent 3’ end features of transcripts influence whether they utilize or bypass the central channel of the RNA exosome [104]. In this context, yMtr4 functions in both direct and channel-based entry routes: it facilitates exosome-channel threading of TRAMP-modified precursors, while also facilitating direct access of snoRNAs and tRNA precursors to the yRrp44-exosome for 3’ processing. The same study also showed that the Ski complex predominantly guides cytoplasmic mRNAs for exosome channel-dependent decay. Moreover, both access routes are involved in the degradation and maturation of RNA Polymerase I-transcribed species, with substrate handover occurring between pathways during processing [104] (Figure 3B).

In human cells EXOSC10 and MTR4 can simultaneously associate with the EXO9 core, wherein the RNA-tethered MTR4 remodels the exosome in a manner that dissuades its association with the EXOSC10 catalytic module and its cofactor C1D, thereby biasing substrate targeting to DIS3 [23] (Figure 3B). Consistent with this, subsequent work identified MPP6 as a central adaptor that bridges MTR4 with the exosome, preferentially stimulating DIS3 activity, with EXOSC10 contributing to a lesser extent, during degradation of polyadenylated transcripts [123]. While yMpp6 associates with the yeast nuclear RNA exosome to function in RNA surveillance, an adaptor role in coordinating yMtr4 and the exosome interaction, as described in metazoans, has not been clearly elucidated [25,124]. In yeast, exosome entry route selection and nuclease engagement appear to be dictated by substrate fate, such that RNAs destined for trimming and maturation are predominantly targeted to the cap-engaged yRrp6, whereas those committed for processive decay are threaded through the channel to yRrp44 [27]. It is noteworthy that yRrp6 can also promote the 3’ processing of 5.8S rRNA and snoRNAs, and the degradation of a subset of rRNA intermediates, in an RNA exosome-independent manner [125].

### 3.4. Cytoplasmic RNA surveillance: entry routes, targets, and coupling to translation

In the cytoplasm, the general mRNA turnover predominantly occurs through deadenylation-coupled decapping, followed primarily by 5’-3’ XRN1-dependent exonucleolysis. In parallel, the 3’-5’ Ski-RNA exosome machinery contributes to the maintenance of the cytoplasmic transcriptome hygiene in specific quality-control scenarios, such as Non-Stop Decay (NSD), No-Go Decay (NGD), and to a lesser extent nonsense-mediated decay (NMD). Studies across several species highlight that the activity of the cytoplasmic RNA exosome is coupled to translation [126–128]. A single-molecule imaging study showed that active translation destabilizes mRNA [129]. Moreover, stretches of non-optimal codons can hamper ribosome translocation rates, increasing the rates of ribosome collisions, which in turn can trigger mRNA decay [130,131]. This intimate coupling between translation and decay allows surveillance machineries to sense the ribosome behaviour and selectively remove defective transcripts without compromising translation overall.

*In vitro* studies have identified a small pool of cytoplasmic DIS3 [19] that is capable of targeting circular RNAs (circRNAs) through its PIN endoribonuclease activity, unlike nuclear DIS3 or cytoplasmic DIS3L1 and independent of RNA exosome association [132]. These findings expand the repertoire of RNA substrates regulated by DIS3 beyond the canonical exosome-associated decay pathways.

#### 3.4.1. Ski- and translation-dependent RNA decay

The budding yeast Ski complex interacts with the 80S ribosomes near the mRNA entry site of the exosome on the 40S small ribosome subunit [42,128,133]. Under normal conditions, mRNA translation is not hindered as the Ski complex adopts a closed conformation, in which the RNA unwinding channel of the ySki2 helicase is blocked by a gatekeeping module comprising its N-terminal domain, ySki3 and two β-propeller subunits of ySki8 [42,134]. Upon translation stalling during NSD and NGD, this ySki2 autoinhibition is relieved, the mRNA is extracted in an ATP-dependent manner in 3’-5’ direction and directly delivered to the exosome [128]. In yeast, this is facilitated by the GTP-binding protein ySki7p that bridges the cytoplasmic exosome [39,40] to the Ski complex by its interaction with ySki3 [41,135]. The C-terminal GTPase domain of ySki7p contributes to ribosome dissociation during decay [136].

In humans, HBS1L3, also referred to as SKI7, a shorter alternatively spliced isoform of *HBS1L*, performs an analogous function by bridging the exosome and engaging the 40S ribosome subunit through the gatekeeping module structurally analogous to Ski2_N-_Ski3-Ski8 in yeast [41]. The Ski2_N-_Ski3-Ski8 module associates preferentially with collided ribosomes, triggering additional ribosome-linked quality control pathways, such as ubiquitination and proteasomal degradation [137]. Thus, translation defects perceived via collision and stalling of ribosomes can simultaneously trigger parallel pathways for defective mRNA and polypeptide turnover [138]. Notably, SKIV2L/ySki2 itself is unable to resolve stalled ribosomes but acts downstream by promoting the degradation of associated defective mRNA species [139]. In contrast, the ribosome-linked quality control factor Pelota (Dom34 in budding yeast) interacts with the empty A site of stalled ribosomes facilitating their release to minimize proteotoxicity.

In yeast, RNAs destined for NMD are primarily decapped and degraded by 5’-3’ exonucleolysis. Although earlier studies suggested a contribution of exosome-mediated 3’-5’ decay [140,141], this likely represents a minor, context-dependent pathway. Similarly, in metazoans, exonucleolytic degradation during NMD is predominantly mediated by XRN1, with lesser contribution from the RNA exosome [142]. One context where exosome engagement during NMD is described is when a subset of RNA substrates are uridylated by TUT4/7 enzymes and degraded by DIS3L1-containing cytoplasmic exosome assemblies [143].

#### 3.4.2. Translation-independent RNA turnover

In addition, to translation-coupled RNA decay, the Ski-exosome pathway also contributes to substrate turnover in a translation-independent manner through Ski-associated component 1 (Ska1), a recently identified subunit of the Ski complex in budding yeast that functions by antagonizing Ski-ribosome association [144]. In this context, the loss of *Ska1* leads to the accumulation of RNA intermediates, including weakly translated mRNAs with longer 3’ UTRs and lncRNAs.

## 4. Regulation of RNA exosome localization and activity

### 4.1. Subcellular localization

The EXO9 core associates with distinct catalytic subunits in different cellular compartments, with its localization and retention being dynamically modulated. Biochemical analyses in budding yeast detected the RNA exosome assemblies both in the nucleus and the cytoplasm [145]. Studies examining yRrp44 and exosome-core proteins yRrp41 and yRrp43 showed that they are predominantly nuclear with nucleolar enrichment [146]. Recent studies have confirmed a predominantly nucleolar localization of most exosome subunits in association with TRAMP complex [147]. This study supports the idea that the cytoplasmic retention of the RNA exosome is mediated by ySki7p. Whether nuclear import of the exosome occurs as a pre-assembled complex, subcomplexes or following the import of individual subunits is not well-understood. However, yRrp6 and yRrp44 have been shown to be imported into the nucleus independent of each other and other core exosome subunits [146]. Recent studies further revealed that the nuclear import of exosome subunits is mediated by karyopherins ySrp1-yKap95 importinα/β heterodimer through redundant import pathways [147].

In humans, DIS3 is mainly nuclear and is excluded from the nucleolus, where EXOSC10 is enriched. Using biochemical analysis in human cells and zebrafish, UTP3, a conserved protein required for rRNA maturation and SSU processome function, was shown to be critical for the nucleolar localization of EXOSC10 and a subset of the SSU subunits [148]. Quantitative nucleolar proteomics uncovered human MTR4 as a major regulator of exosome translocation and retention into the nucleolus [149]. Finally, this study found exosome localization to be sensitive to the stability of individual subunits.

### 4.2. Posttranslational modifications and RNA exosome activity

Beyond cellular compartmentalization, the activity of the RNA exosome is also regulated by a range of posttranslational mechanisms (PTMs) including acetylation, phosphorylation and SUMOylation that govern subunit stability, trafficking, and localization. Proteomic characterization of the RNA exosome identified phosphorylation sites on multiple subunits and cofactors, including yCsl4, yRrp4, yRrp43, yMtr3, yRrp6 and ySki7 [150,151]. In *S. pombe*, PTM profiling of the purified exosome revealed 39 modified sites encompassing acetylation, methylation and phosphorylation, on several RNA exosome subunits, including yRrp44, and functional analysis confirmed an essentiality role of these PTMs in fine-tuning RNA exosome activity [152]. The yRrp6 is SUMOylated [153], consistent with this, human EXOSC10 was shown to undergo SUMOylation in response to cold exposure which suppressed its abundance [154]. A subsequent study revealed that USP36, a nucleolar ubiquitin-specific protease, binds EXOSC10 and is necessary for its SUMOylation at the lysine (K) 583 residue, which is important for nucleolar exosome function during ribosome biogenesis [155]. Further work showed that hypoxia triggers deSUMOylation of EXOSC10, accompanied by its dissociation from USP36 and its translocation from nucleolus to the nucleoplasm, in a manner independent of the hypoxia-independent factor (HIF); this affected the mRNAs with no detectable effect on rRNA maturation [156]. Together, these findings support a model wherein the PTMs on exosome subunits serve as adaptive molecular switches integrating environmental and physiological cues with the modulation of RNA degradation machineries and therefore the transcriptome.

## 5. RNA exosome associated human diseases

The dysregulation of the RNA exosome is implicated in a plethora of human diseases congruent with its broad relevance to human pathophysiology. Here, we limit our focus to monogenic diseases and complex diseases, such as cancers.

### 5.1. RNA exosome-linked monogenic diseases

Although the RNA exosome is ubiquitously expressed, many RNA exosome-linked disorders manifest with selective organ-system involvement, underscoring the tissue-specific vulnerability to impaired RNA decay machineries. Pathogenic variants in several genes encoding different subunits of the EXO9 scaffold cause predominantly neurological phenotypes within the spectrum of pontocerebellar hypoplasia type 1 (PCH1). Homozygous mutations in *EXOSC1*, *EXOSC3, EXOSC8* and *EXOSC9* are associated with PCH1 subtypes, namely PCH1F (OMIM #619304), PCH1B (OMIM #614678), PCH1C (OMIM #616081), and PCH1D (OMIM #618065), respectively. These disorders are marked by underdevelopment of the cerebellum, global developmental delay, hypotonia and variable motor neuron involvement [157–160]. In contrast, disease-causing variants in other subunits can result in phenotypes with multisystemic involvement. For example, *EXOSC2* biallelic variants cause a distinct neurological disorder termed short stature, hearing loss, retinitis pigmentosa, distinctive facies syndrome (SHRF; OMIM# 617,763). Similarly, homozygous pathogenic variants in *EXOSC5* cause a multisystemic disorder termed cerebellar ataxia, brain abnormalities and cardiac conduction defects (CABAC; OMIM #619576). Its clinical features reflect how the effects of RNA surveillance disruption extend beyond neurodevelopment to broadly impair ribosome biogenesis and tissue homoeostasis. This contrasts with PCH, which is primarily caused by early neurodevelopmental defects. Beyond exosome core subunits, variants in the NEXT subunit ZCCHC8 have been linked to telomere-related pulmonary fibrosis and/or bone marrow failure (OMIM #618674), highlighting the importance of RNA surveillance efficiency in maintenance of genomic stability and stem-cell function. Pathogenic variants in SKI subunits, SKIV2L/SKIC2 and TTC37/SKIC3 are associated with trichohepatoenteric syndrome 2 (OMIM #614602) and trichohepatoenteric syndrome 1 (OMIM #222470), respectively. These disorders are multisystemic, characterized by severe intrauterine growth retardation. Intractable diarrhoea, hair abnormalities, liver dysfunction and immunodeficiency, underscoring the importance of cytoplasmic RNA surveillance in epithelial, intestinal, hepatic and developmental homoeostasis. Table 1 summarizes canonical RNA exosome and selected cofactor subunits, their orthologs across commonly used model organisms, and their established and emerging human monogenic disease associations.

**Table 1.** List of RNA exosome and selected cofactor subunits, molecular functions, orthologs in mouse, zebrafish, *Drosophila* and budding yeast, and human monogenic disease associations.

Human gene	Function	Orthologs				Disease associations with established or emerging monogenic relevance	OMIM #
		Mouse	Zebrafish	Drosophila	Budding yeast		
*EXOSC1*	EXO9 cap subunits RNA binding and substrate recognition	*Exosc1*	*exosc1*	*Csl4*	*Csl4*	PCH1F	619304
*EXOSC2*		*Exosc2*	*exosc2*	*rrp4*	*Rrp4*	Short stature, Hearing loss, Retinitis Pigmentosa and Distinctive Facies (SHRF)	617763
*EXOSC3*		*Exosc3*	*exosc3*	*Rrp40*	*Rrp40*	PCH1B	614678
*EXOSC4*	EXO9 barrel scaffold containing channel for RNA substrate	*Exosc4*	*exosc4*	*Ski6*	*Rrp41/Ski6*	Single family with neurodevelopmental disorder and renal failure [1]	–
*EXOSC5*		*Exosc5*	*exosc5*	*Rrp46*	*Rrp46*	Cerebellar ataxia, brain abnormalities and cardiac conduction defects (CABAC)	619576
*EXOSC6*		*Exosc6*	*exosc6*	*Mtr3*	*Mtr3*	–	–
*EXOSC7*		*Exosc7*	*exosc7*	*Rrp42*	*Rrp42*	–	–
*EXOSC8*		*Exosc8*	*exosc8*	–	*Rrp43*	PCH1C	616081
*EXOSC9*		*Exosc9*	*exosc9*	*Rrp45*	*Rrp45*	PCH1D	618065
*DIS3*	nuclear 3’-5’ exo- and endoribonuclease	*Dis3*	*dis3*	*Dis3*	*Rrp44*	Two unrelated families with primary ovarian insufficiency [2,3]	–
*DIS3L1*	cytoplasmic 3’-5’ exoribonuclease	*Dis3l1*	*dis3l1*				
*EXOSC10*	nucleolar exoribonuclease	*Exosc10*	*exosc10*	*Rrp6*	*Rrp6*	–	–
*MTR4/SKIV2L2*	RNA helicase, component of TRAMP-like, NEXT and PAXT complexes	*Mtr4*	*mtrex*	*Mtr4*	*Mtr4*	–	–
*SKIV2L/SKIC2*	RNA helicase, cytoplasmic SKI complex component	*Skiv2l*	*skiv2l*	*twister*	*Ski2*	Trichohepatoenteric syndrome 2	614602
*TTC37/SKIC3*	SKI complex scaffold	*Ttc37*	*skic3*	*ski3*	*Ski3*	Trichohepatoenteric syndrome 1	222470
*RBM7*	RNA-binding component of NEXT	*Rbm7*	*rbm7*	CG11454	–	Spinal muscular neuropathy [4]	
*ZCCHC8*	scaffold subunit of NEXT	*Zcchc8*	*zcchc8*	–	*dZcchc8*	Pulmonary fibrosis and/or bone marrow failure	618674

PCH: pontocerebellar hypoplasia.

OMIM: Online Mendelian Inheritance in Man.

### 5.2. RNA exosome dysfunction in cancers

DIS3 is strongly implicated in haematological malignancies, such as multiple myeloma (MM). Heterozygous chromosomal deletions spanning DIS3 locus, recurrent somatic and inherited germline DIS3 mutations that are mainly missense are noted in up to 18% MM supporting its role as germline susceptibility and driver gene in plasma cell malignancies [161–164]. Mechanistic studies reveal that DIS3-mutated MM cases exhibit a characteristic transcriptional profile consistent with dysregulation of RNA metabolism, including upregulation of long ncRNA [165,166]. Functional studies support its tumour suppressor function as DIS3 loss causes genomic instability via the genome-wide accumulation of DNA:RNA hybrids (R-loops) that impaired homologous recombination-based DNA repair, increased DNA damage and triggered interferon response and exacerbated mutational burden in MM tumour cells [167]. Further studies highlighted an essential role of DIS3 in regulating B cell proliferation, physiological and malignant differentiation of plasma cells in humans [168]. Beyond MM recurrent DIS3 mutations are also reported in acute myeloid leukaemia [169] suggesting that haematopoietic lineages may be particularly sensitive to DIS3-dependent RNA surveillance.

Beyond DIS3 altered expression of other RNA exosome subunits, including EXOSC10 [170,171] and EXOSC4 [172] have been reported in several types of malignancies.

## 6. Model organisms in RNA exosome biology

### 6.1. Budding yeast

In budding yeast (*Saccharomyces cerevisiae)*, all EXO9 subunits are essential for viability [7,173]. Among the cap subunits, yRrp4, yRrp40 and yCsl4 are necessary for RNA substrate-binding, and additionally yCsl4 mediates unique cofactor-binding interactions [15]. Among the catalytic subunits, yRrp6 is not essential, but its deletion exacerbates the accumulation of multiple classes of aberrant RNAs, including those unprocessed, resulting in slower growth and enhanced temperature sensitivity [2,96,174].

Studies in yeast modelling monogenic RNA exosomopathies included validated and emerging candidate RNA exosome-disease associations by the introduction of patient-derived variants in *EXOSC1* [175], *EXOSC2* [176–178], *EXOSC3* [177,179], *EXOSC4* [178,180], *EXOSC5* [177,181] and EXOSC9 [178] in their orthologous *S. cerevisiae* genes. This produced loss of viability or attenuated growth of mutant yeast. Comparative transcriptomic analyses of mutant *yRrp4*, *yRrp40* and *yRrp46* yeast revealed overlapping but distinct transcriptomic alterations, particularly impeding ribosome biogenesis and translation, suggesting that individual RNA exosome subunits may differ in their *in vivo* contribution to RNA surveillance [177].

### 6.2. Fruit fly

In fruit fly (*Drosophila melanogaster)*, all EXO9 subunits are conserved except for EXOSC8 [182]. The *Drosophila* nuclease dDis3 performs both nuclear and cytoplasmic exosomal activities [183], and has been detected in the nucleoplasm, nuclear periphery and the cytoplasm, with its distribution being coordinated with that of dRrp6 [184].

*Drosophila* genetics has been extensively leveraged to investigate RNA exosome-linked disease mechanisms. Johnstone *et al.*, proposed compound heterozygous mutations in *DIS3* as the underlying genetic cause of primary ovarian insufficiency (POI) in two sisters [185]. In this study, *dDis3* knockdown (KD) in *Drosophila* ovaries led to complete infertility. These findings were supported by a subsequent study reporting a biallelic missense mutation in *DIS3* in an unrelated POI patient [186]. Overexpression of this mutant allele in *Drosophila* impaired ovarian development and caused egg chamber degeneration. Furthermore, this study showed that *dDis3* KD in the female germline and somatic gonadal precursor cells resulted in dysgenic ovaries and complete loss of ovaries, respectively. Consistent with a broader role of the RNA exosome in oogenesis, germline depletion of *dDis3*, *dRrp6*, the Integrator subunit *dIntS1* and NEXT adaptors d*ZC3H18* and *dArs2* led to improper accumulation of transcripts during *Drosophila* oogenesis, and in fly-derived cell lines [187].

The ablation of *Rrp4*, the *Drosophila* ortholog of *EXOSC2*, which is linked with SHRF was lethal, with rare escapers exhibiting eye disorganization and abnormalities in muscle ultrastructure, and wing vein development [188]. In this study, the ubiquitous *dRrp4* KD was lethal, whereas its selective depletion in the eye produced small and rough eyes, with impaired autophagy machinery shown to contribute to the SHRF phenotypes both in *Drosophila* and *EXOSC2*-mutant patient cells.

To model PCH1B, knockdown of *dRrp40*, the *Drosophila* ortholog of *EXOSC3*-associated with PCH1B was performed ubiquitously and in a tissue-specific manner, in the glia, neurons and muscle, which was lethal [189]. In the same study, *dRrp40* conditional depletion led to age-dependent neuronal phenotypes in adult flies. Further, they used CRISPR/Cas9 genetic engineering to introduce two *EXOSC3* patient-linked variants in *dRrp40*, which reduced steady-state protein levels of multiple RNA exosome subunits and caused transcriptome anomalies in the fly-brain, including altered abundance of genes essential for neuronal function, with concomitant reduction in survival and progressive neurological defects. A subsequent study analysed the CRISPR/Cas9 edited flies harbouring *EXOSC3* pathogenic variants in *dRrp40* by single-nucleus RNA sequencing to reveal broad transcriptomic dysregulation, including that in rRNA processing in brain-enriched cell-populations, which correlated with attenuated survival, neurodegeneration and behavioural anomalies congruent with severity of the mutation [190]. Together, these studies for causal testing of human disease alleles in *Drosophila* reveal how disruption of RNA surveillance can translate into neurodevelopmental and reproductive pathology in vivo.

### 6.3. Zebrafish

Zebrafish (*Danio rerio*) has emerged as a powerful vertebrate system both for the mechanistic dissection of RNA exosome-related pathways, and for *in vivo* disease modelling of RNA exosomopathies. Shan *et al.*, (2023) investigated Urb1, a conserved nucleolar protein essential for 60S ribosomal subunit maturation in zebrafish. Urb1protein binds pre-ribosomal particles and participates in 3′-ETS removal from nascent pre-rRNA transcript, thereby acting as a scaffold that coordinates the recruitment of ribosome-biogenesis factors [191]. In *urb1*-deficient zebrafish, early steps of pre-60S maturation collapse, resulting in the aberrant accumulation of 3’ETS-containing pre-rRNA-intermediates. This activates the nucleolar exosome – mediated surveillance, ultimately suppressing mature 28S rRNA production, and leads to defective craniofacial and head development.

A study examining Ski adaptor function in zebrafish development found that *ski7* mRNA expression peaks during the oocyte-to-embryo transition [192]. In this study, genetic ablation of *ski7* severely compromised egg quality, whereas its depletion post the single-celled stage produced no overt defects, indicating that Ski7 primarily functions during oogenesis in zebrafish. Transcriptome analysis in this study revealed that Ski7 functions preferentially in the turnover of low-abundance maternal transcripts. Moreover, the *ski7*^*-/-*^ zebrafish displayed increased resistance to reductive stress, supporting a role for cytoplasmic exosome-mediated RNA surveillance in redox-sensitive pathways during early development.

PCH1B linked to *EXOSC3* was the first RNA exosome-linked disorder modelled in zebrafish and the *exosc3* morphants exhibit small brains, collapsed hindbrain and loss of cerebellar Purkinje neurons, leading to their early demise, thereby closely recapitulating the PCH1B patient phenotypes [157]. Subsequent zebrafish models of mutations in other exosome subunits revealed similarly profound neurodevelopmental abnormalities, for example *exosc8* morphants [159], *exosc9* morphants and null crispants [158] exhibit severe cerebellar and hindbrain abnormalities, and motor-neuron dysfunction, paralleling the clinical features of human patients with PCH1C and PCH1D, respectively. Giunta *et al.*, (2016) generated *exosc3*, *exosc8* and *rbm7* morphants that showed convergent phenotypes with motor neuron and cerebellar defects, emphasizing the vulnerability of vertebrate neurodevelopment to RNA metabolism [193]. Further, *rbm7* morphants had brain and motor neuron defects mirroring the clinical phenotypes in *RBM7*-associated spinal-motor neuropathy, and closely phenocopied *exosc3*, *exosc8* and *exosc9* mutant zebrafish features [158].

At the molecular level, homozygous *exosc8* and *exosc9* crispants exhibit upregulation of p53 responsive and ribosome biogenesis transcripts, G2/M cell-cycle arrest, and excess of apoptosis in the developing brain, leading to decreased cerebellar and head size [194]. Similarly, *exosc5*^*-/-*^ crispants recapitulate midbrain and cerebellar defects characteristic of *EXOSC5*-associated CABAC [181]. The *exosc2*^*-/-*^ crispants showed abnormal myelin related transcripts and an imbalance of the nucleotide pool, concomitant with microcephaly, hypomyelination, microphthalmia, retinitis pigmentosa and early larval lethality [195], that are consistent with the retinal and neurodevelopmental abnormalities noted in *EXOSC2*-related SHRF [196]. Together, these studies support the important role of the RNA exosome at the intersection of global RNA homoeostasis and p53-mediated neuronal apoptosis during vertebrate cerebellar and hindbrain development.

### 6.4. Mouse

Only a subset of the RNA exosome subunits have been interrogated genetically in mice (*Mus musculus)*. Among EXO9 cap subunits, *Exosc1* and *Exosc2* null mice are embryonic lethal, albeit at distinct developmental stages [197]. While, *Exosc1*^*-/-*^ mice embryos form an egg cylinder, they fail to initiate gastrulation and arrest prior to primitive streak formation, the *Exosc2* null embryos develop to morphologically normal blastocyst till embryonic day 3.5 and succumb during the peri-implantation stages, which is earlier than the stage of lethality observed in *Exosc1* null mice. Subsequent work delineated a role for Exosc2 in the development and maintenance of the inner cell mass. To date, no ubiquitous *Exosc3* null mouse has been reported. However, its specific depletion in mice B-cells caused an early blockade in differentiation, together with the accumulation of ncRNAs and activation of p53 pathway [198]. *Exosc3* conditional depletion in mouse ESCs and B-cells also resulted in dramatic enrichment of eRNAs and lncRNAs, exacerbating R-loop-associated DNA double-stranded breaks [3,199], highlighting the essential role of RNA surveillance in maintaining genome stability.

Consistent with the crucial role of the RNA exosome in mouse development, the *Exosc10* null mice embryos exhibit embryonic lethality, arresting at the eight-cell embryo-to-morula transition stage prior to blastocyst formation [200]. Tissue-specific studies have further clarified Exosc10 function in mammalian germline development. Oocyte-specific ablation of *Exosc10* during the growth phase impaired rRNA processing and dysregulation of multiple RNA classes, including mRNAs encoding for vesicular trafficking and meiotic cell cycle regulators, attenuating growth phase-to-maturation transition required for oocyte quality maintenance, thereby producing developmentally incompetent oocytes and female subfertility [201]. The conditional inactivation of *Exosc10* at an earlier primordial follicle stage caused severe defects to oocyte development and maturation, resulting in ovarian reserve depletion and complete female infertility [202]. Similarly, conditional deletion of *Exosc10* in male meiotic germ cells disrupted spermatogenesis, resulting in reduced sperm count and male subfertility [203]. Subsequent studies showed that *Exosc10* ablation in the male germline produced widespread transcriptomic dysregulation, concomitant with impaired meiotic progression, compromised spermatogenesis and complete male infertility [204].

The global knockout of *Dis3* arrested mouse embryonic development in preimplantation stage during the morula-to-blastocyst transition causing early lethality [205]. Mechanistically, this study showed that Dis3 controls cell-fate specification by degrading mRNAs encoding for *Pou6f1*, a transcriptional repressor that controls the expression of cell-lineage defining regulators, such as *Nanog* and *Cdx2*. Oocyte-specific inactivation of *Dis3* led to accumulation of pervasive transcripts, and elevated levels of the repressive H3K27me3 modification, which sequestered Pol II and blocked meiotic progression. This study also demonstrated that the combined ablation of *Dis3* and *Exosc10* exacerbated the phenotype, producing earlier oocyte growth defects and enhanced accumulation of intergenic RNAs. Studies also reveal that Dis3 is required for male fertility in mouse. Its conditional deletion in the male germline caused enrichment of pervasive transcripts, impaired spermatogenesis, and resulted in Sertoli-cell-only phenotype with complete male sterility [206]. Together, mice models for various RNA exosome components underscore the importance of precise RNA surveillance in early mammalian embryogenesis, cell-fate specification, genome integrity and germline development.

## 7. Conclusion

The RNA exosome has emerged as a focal hub that coordinates RNA homoeostasis and transcriptome fidelity across nuclear and cytoplasmic compartments. In this review, we highlight how it engages diverse classes of RNA substrates, and how its functional specificity arises not only from the dynamic regulation of its subunits, but also that of several cofactor assemblies that interface with pervasive transcription, promoter-proximal pausing and transcriptional termination. The modular organization of the RNA exosome and broad range of its regulatory mechanisms underscore its relevance as an active modulator of the architecture of gene expression, rather than a passive disposal unit for unnecessary or defective transcripts. The expanding spectrum of RNA exosomopathies shed light on its critical role in development and physiology, while disparate disease phenotypes reveal tissue-specific vulnerabilities to impaired RNA surveillance. Insights from budding yeast, *Drosophila*, zebrafish and mouse models concur that the role of the RNA exosome is cell-type and context-dependent despite its ubiquitous expression. Across model-organisms, RNA exosome dysfunction disrupts developmental programmes, morphogenesis and cellular homoeostasis, offering mechanistic insights into pathomechanisms underlying RNA exosome-associated diseases. Taken together, these findings position the RNA exosome as a dynamic regulatory node in safeguarding transcriptome integrity, advancing our understanding of fundamental RNA biology and its relevance to human disease.

## 8. Future perspectives

Despite substantial progress, key conceptual gaps remain in our understanding of RNA exosome biology. An important challenge is to resolve how individual substrates are selectively routed to distinct arms of the RNA exosome, assisted by distinct cofactors, such as TRAMP, NEXT, PAXT, and SKI, or via early RNA surveillance and translation-dependent quality-control mechanisms, involving Integrator/Restrictor-mediated termination, and ribosome-coupled decay, respectively. Addressing this will require high-resolution structural and biochemical strategies to elucidate how RNA substrate features and cofactor engagement are coupled to nuclease selection, thereby intricately mapping distinct exosome decay pathways.

An increasing appreciation of pervasive transcription, promoter directionality, and heterogeneity in transcription termination strategies underscores the need to examine how the RNA exosome actively sculpts the gene expression landscape, particularly during development and morphogenesis. Combining CRISPR/Cas9 mediated gene editing at defined exosome target loci together with nascent RNA sequencing, long-read transcript profiling, and chromatin-contact mapping, may help to elucidate how RNA turnover influence transcriptional boundaries, promoter directionality and enhancer prioritization at different genomic contexts.

The expanding catalogue of RNA exosome-linked diseases underscores the urgent need to investigate RNA exosome function in disease-relevant tissues, particularly the brain and central nervous system. Integrating single-nucleus transcript sequencing with patient-derived induced pluripotent stem cells and organoids will offer deeper insights into tissue-specific vulnerabilities to RNA exosome dysfunction and facilitate the identification of genetic modifiers of disease phenotypes. Humanized animal models generated through gene-editing will aid in mapping the conserved RNA decay pathway modulating the transcriptome in different tissues and developmental stages. Together, this may reveal actionable molecular entry-points that may be leveraged for therapeutic targeting of the RNA exosome function.

## Data Availability

All data in this review are publicly available.
